# Supplementary material for: Crab-Eating Monkey Acidic Chitinase (CHIA) Efficiently Degrades Chitin and Chitosan under Acidic and High-Temperature Conditions
Source: Molecules. 2022 Jan 9;27(2):409. doi: 10.3390/molecules27020409 (PMC8781735; doi:10.3390/molecules27020409)
Supplement: Supplementary file 1 [file molecules-27-00409-s001.zip › molecules-1539649-supplementary.pdf]

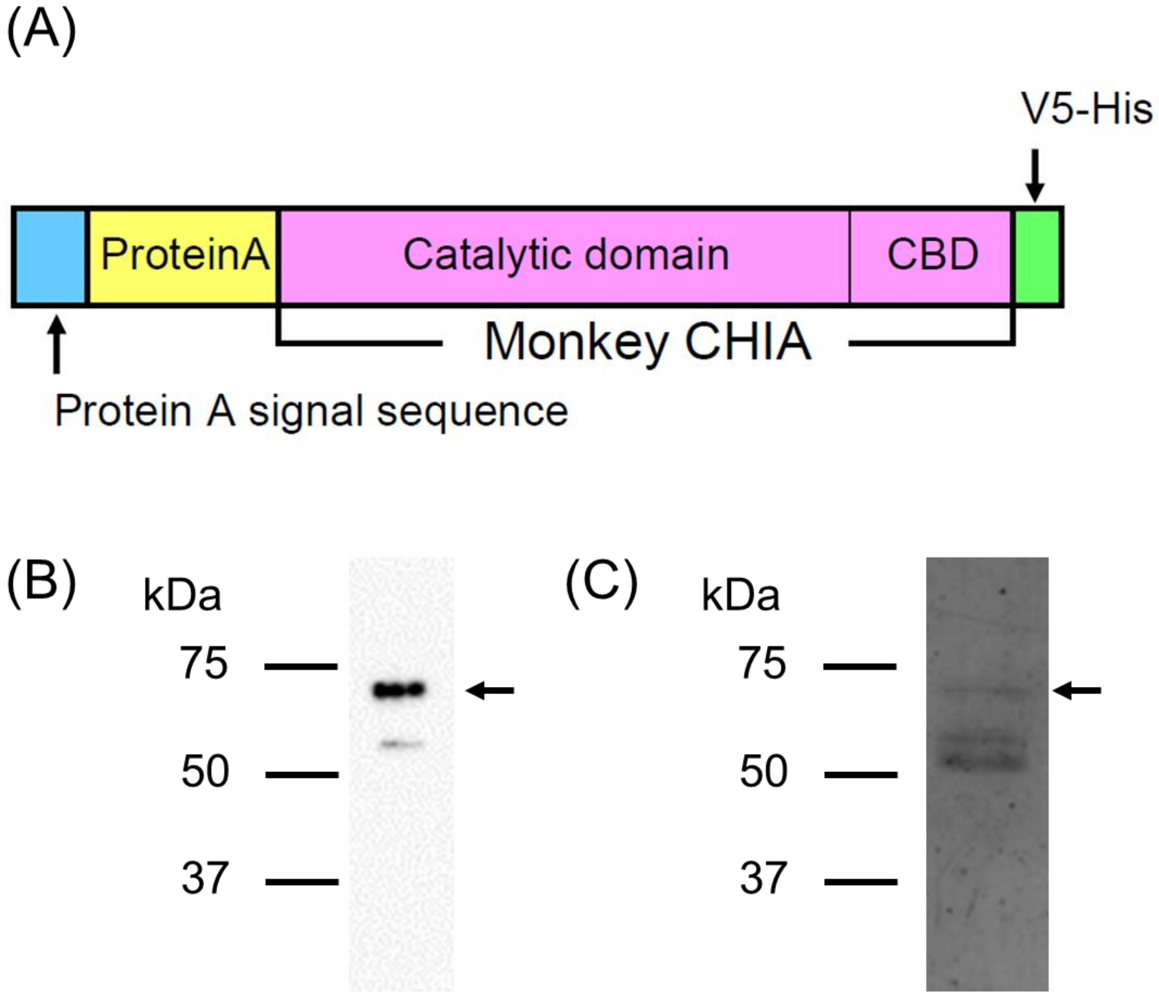

Supplementary Figure S1. Expression of monkey CHIA in *E. coli* as fusion protein.

(A) Schematic representation of the E. coli-expressed monkey CHIA fusion protein (Protein A-monkey CHIA V5-His). The estimated size for Protein A-monkey CHIA-V5-His is 68 kDa. (B) Western blot analysis of the recombinant proteins using anti-V5 antibody. (C) SYPRO Ruby staining.

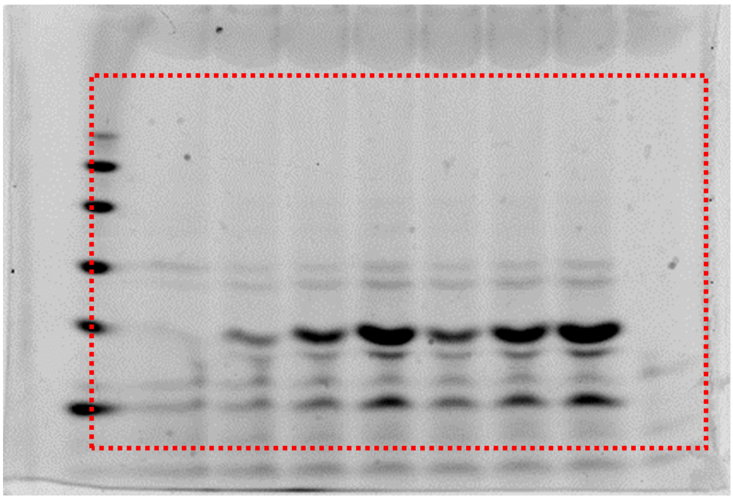

Supplementary Figure S2. Full-length gel shown in Figure 1A. Full-length gel image of FACE method in Figure 1A.

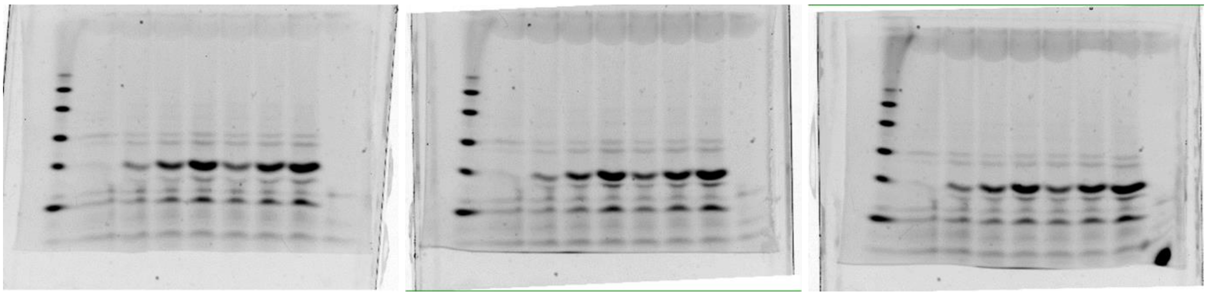

Supplementary Figure S3. Gels for quantitative analysis shown in Figure 1B. Gel images of FACE method in Figure 1A. Signal intensities of $(GlcNAc)_2$ in these gel images were quantified and are shown in Figure 1B.

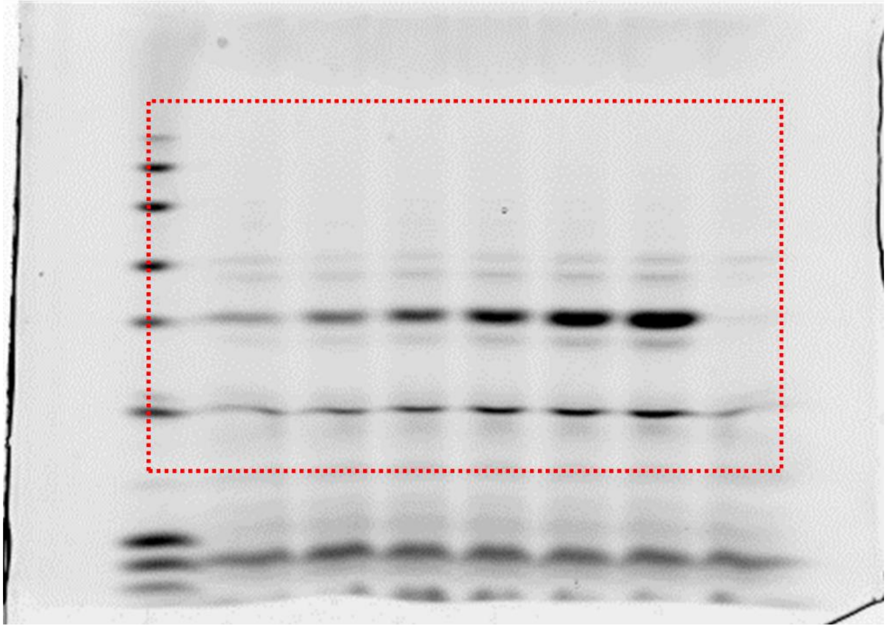

Supplementary Figure S4. Full-length gel shown in Figure 1C. Full-length gel image of FACE method in Figure 1C.

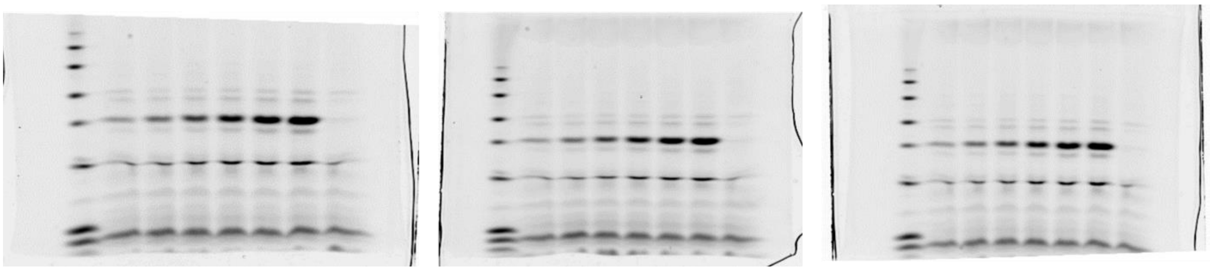

Supplementary Figure S5. Gel for quantitative analysis shown in Figure 1D. Gel images of FACE method in Figure 1C. Signal intensities of $(GlcNAc)_2$ in these gel images were quantified and are shown in Figure 1D.

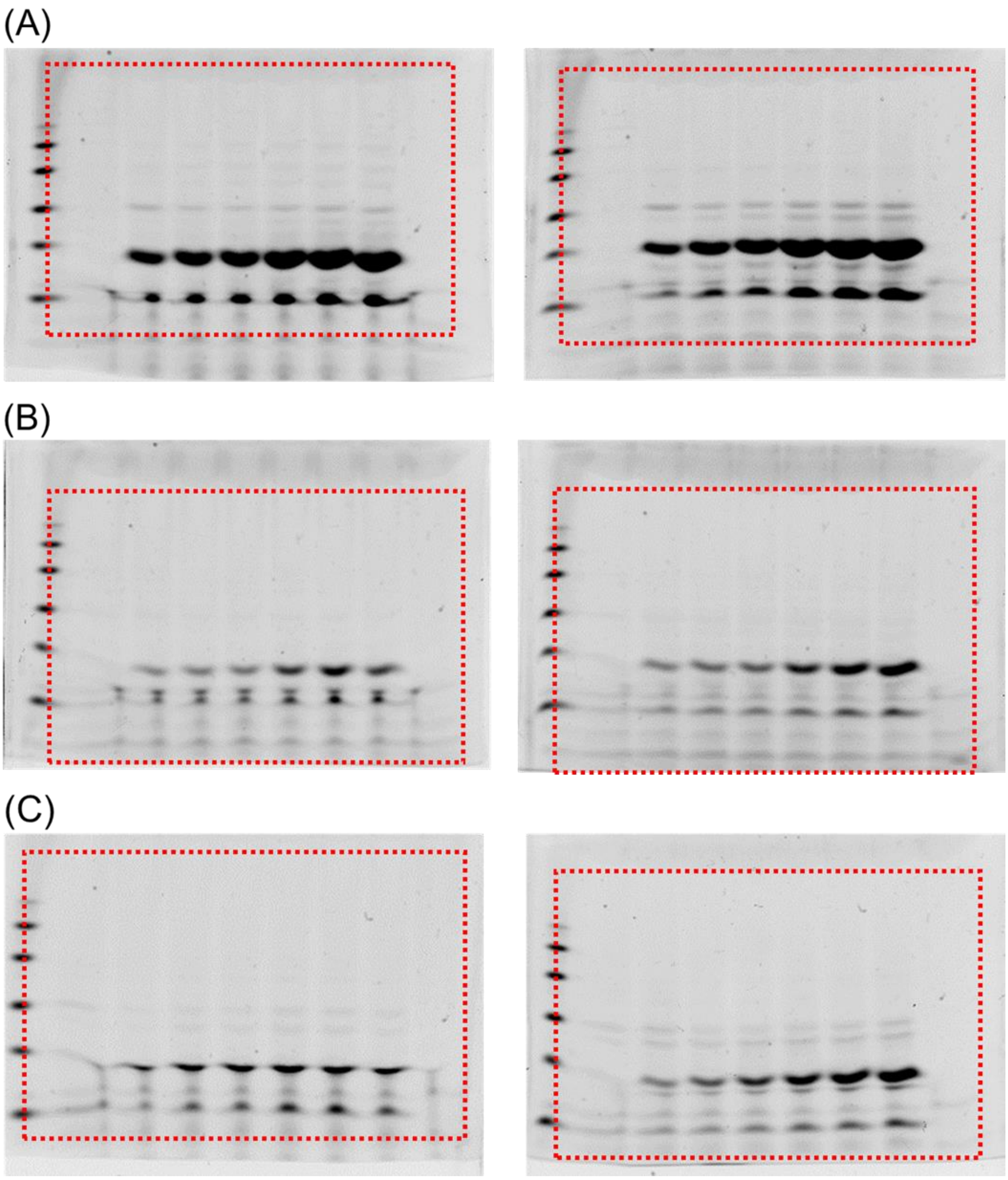

Supplementary Figure S6. Full-length gel shown in Figure 2. Full-length gel images of FACE method in Figure 2. (A) P-chitin, (B) colloidal chitin and (C) α-chitin. pH 2.0 (left) and pH 5.0 (Right).

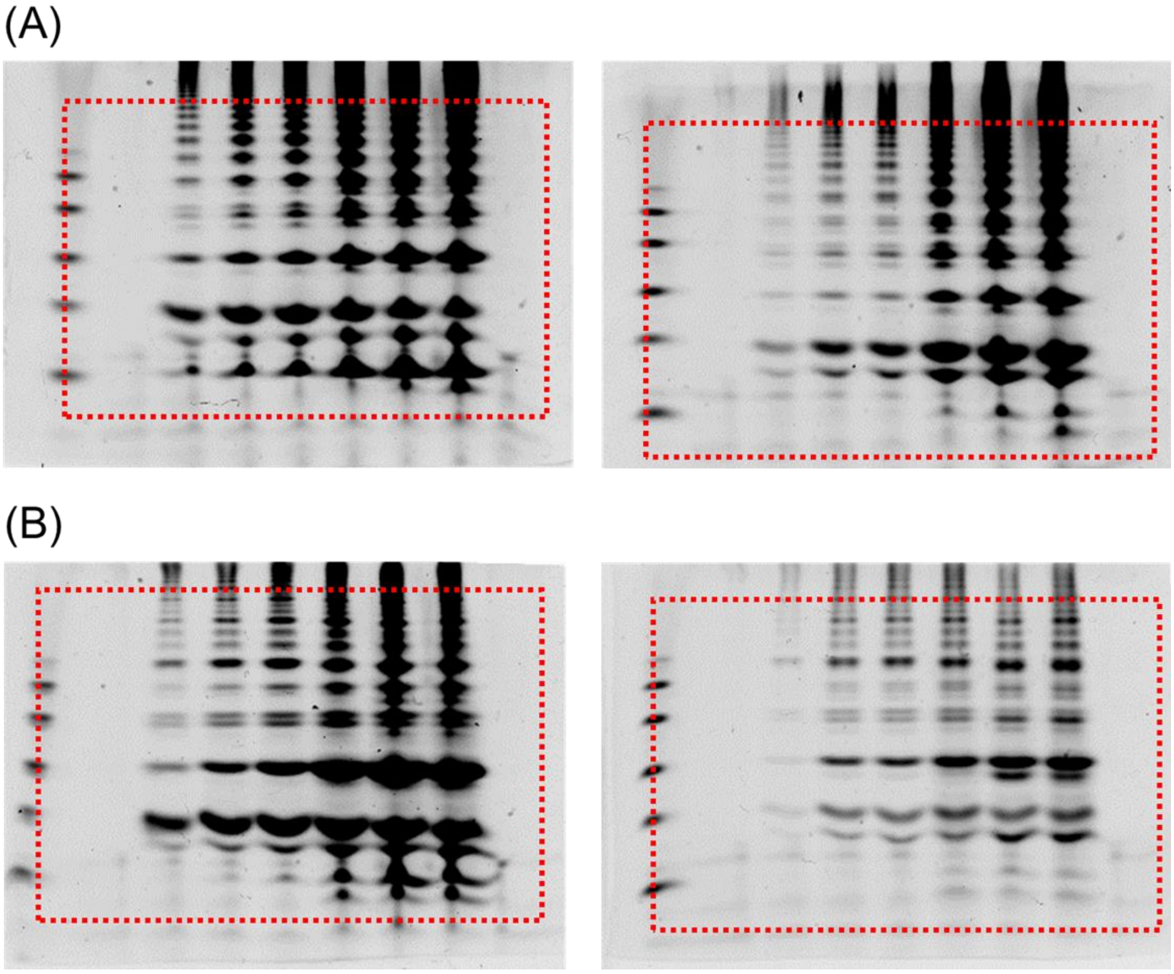

Supplementary Figure S7. Full-length gel shown in Figure 3. Full-length gel images of FACE methods in Figure 3. (A) Random-type chitosan and (B) block-type chitosan. pH 2.0 (left) and pH 5.0 (right).

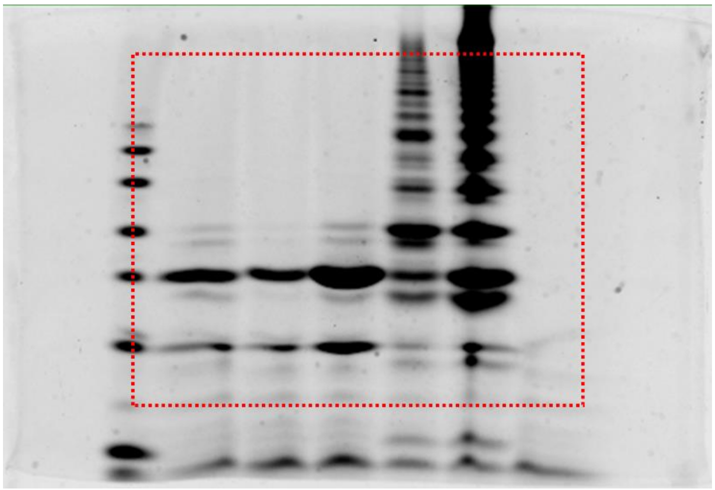

Supplementary Figure S8. Full-length gel shown in Figure 4A. Full-length gel image of FACE method in Figure 4A.

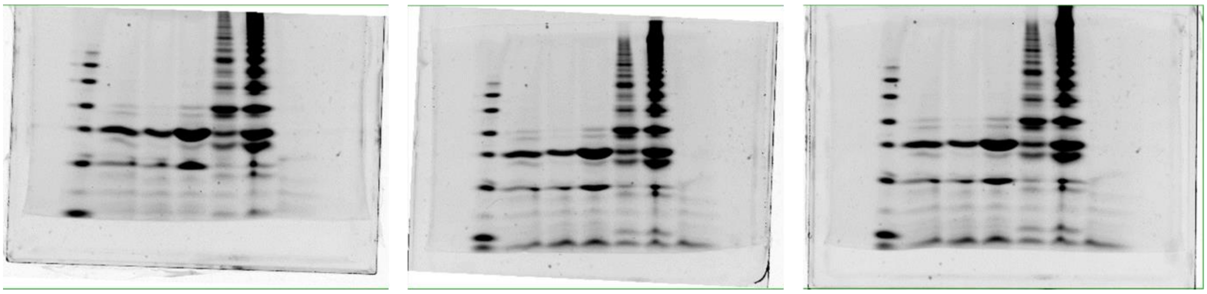

Supplementary Figure S9. Gels for quantitative analysis shown in Figure 4B. Gel images of FACE method in Figure 4. Experiments were conducted in triplicate. Signal intensities of (GlcNAc)n (n=2-6) in these gel images are quantified and shown in Figure 4B.

Supplementary Table S1. The quantitative data in Figure 4B.

	$(GlcNAc)_2$	$(GlcNAc)_3$	$(GlcNAc)_4$	$(GlcNAc)_5$	$(GlcNAc)_6$	Total
α-chitin	11.5%	1.4%	—	—	—	12.9%
Colloidal chitin	8.2%	—	—	—	—	8.2%
P-chitin	46.2%	2.1%	—	—	—	48.3%
Block-type chitosan	8.4%	50.6%	4.5%	2.6%	5.7%	71.8%
Rndom-type chitosan	39.0%	17.9%	15.8%	13.3%	14.0%	100.0%
